# Ruptured Suprasellar Dermoid Cyst Treated With Lumbar Drain to Prevent Postoperative Hydrocephalus: Case Report and Focused Review of Literature

**DOI:** 10.3389/fsurg.2021.714771

**Published:** 2021-08-11

**Authors:** Sarah E. Blitz, Joshua D. Bernstock, Adam A. Dmytriw, Daniel Francis Ditoro, Ari D. Kappel, William B. Gormley, Pierpaolo Peruzzi

**Affiliations:** ^1^Harvard Medical School, Boston, MA, United States; ^2^Department of Neurosurgery, Brigham and Women's Hospital, Harvard University, Boston, MA, United States; ^3^Department of Radiology, Brigham and Women's Hospital, Harvard University, Boston, MA, United States; ^4^Department of Pathology, Brigham and Women's Hospital, Harvard University, Boston, MA, United States

**Keywords:** dermoid, cyst, hydrocephalus, rupture, lumbar drain, neurosurgery, intracranial

## Abstract

**Background:** Ruptured intracranial dermoid cysts are extremely rare. Standard treatment consists of endonasal decompression or craniotomy with evacuation and copious irrigation of subarachnoid spaces to remove any disseminated cystic contents. Disseminated fat particles in the subarachnoid space may be the cause of further sequalae, including the subsequent development of chemical meningitis and hydrocephalus. Here, we present a case of ruptured suprasellar dermoid cyst treated with craniotomy for emergent optic nerve decompression, followed by postoperative hydrocephalus successfully treated with lumbar drain.

**Case description:** We describe a 30-year-old man with a history of migraines who presented with acute onset of headache, photophobia, nausea, vomiting, and vision loss in the left eye. Head CT and brain MRI demonstrated a ruptured suprasellar dermoid cyst with associated mass effect on the optic nerves and frontal lobes as well as fat attenuation material within the subarachnoid spaces. The patient underwent left frontotemporal craniotomy for cyst resection and developed non-obstructive hydrocephalus on postoperative day 1, refractory to external ventricular drainage. Placement of a lumbar drain cleared the subarachnoid space of debris derived from the ruptured dermoid cyst, and the hydrocephalus resolved. The patient did not require permanent CSF diversion.

**Conclusions:** Intracranial dermoid cysts are uncommon, and rupture is a rare event. Standard surgical treatment with craniotomy for evacuation may leave disseminated dermoid contents and fat particles throughout the subarachnoid spaces. We highlight a case of ruptured suprasellar dermoid cyst with postoperative communicating hydrocephalus treated with lumbar drain when external ventricular drain (EVD) was ineffective. Review of the current literature reveals inconsistent findings on the effects of remaining fat particles. In cases with clinical evidence of increased intracranial pressure due to non-obstructive hydrocephalus attributable to chemical meningitis, temporary lumbar drainage is an option to be considered before committing the patient to permanent shunting.

## Introduction

Dermoid cysts (mature cystic teratoma) are benign, developmental tumors that arise from inclusion of ectodermal cells at the time of neural tube closure (1). They contain elements of retained hair, teeth, sweat, and sebaceous glands, often with large pockets of fat and cholesterol (2). Intracranial dermoid cysts are typically diagnosed in the 2nd or 3rd decade of life (3), and most commonly occur in the suprasellar space, Sylvian fissure, or posterior fossa including the cerebellopontine angle or within the 4th ventricle (4). Extracranial sites include the spine and orbit, which may occur with fistulous connections to the skin or dermal sinus tract (5, 6). Malignant transformation into squamous cell carcinoma is extremely rare but has been reported (7). Dermoid cysts account for 0.04–0.6% of all intracranial tumors (8), and rupture is a relatively uncommon occurrence accounting for 0.18% of all new central nervous system tumors operated on during a 12-year period at a major tertiary care center (8). They usually occur spontaneously, but cases of rupture secondary to closed head trauma or iatrogenic surgical complications have been referenced (3, 9). The pathophysiology behind spontaneous rupture is not well-understood (3). Rupture can cause serious complications, including chemical meningitis, visual loss, vasospasm, and cerebral infarction. Patients commonly present with headache (32.6%), followed by seizures (26.5%), cerebral ischemia with sensory or motor deficit (16.3%), and aseptic meningitis (8.2%) (10). Symptoms related to mass effect depend on the location of the cyst: suprasellar cysts may present with bitemporal hemianopsia, diplopia, and visual loss; cysts in the sylvian fissure may present with seizures; posterior fossa and 4th ventricular cysts may present with obstructive hydrocephalus or lower cranial nerve findings (8). Rupture results in dissemination of the dermoid contents into the subarachnoid space and/or ventricles (11).

Surgical resection of the remaining cystic contents and tumor capsule is the mainstay of treatment. Fat droplets within the subarachnoid space are often visualized in ruptured cases and can be removed with suction and irrigation. However, disseminated cystic contents, including fat droplets, inevitably remain. Due to the tumor rarity and wide range of postoperative outcomes, the impacts of the remaining fatty deposits are unclear. In this report, we describe a case of ruptured suprasellar dermoid cyst with subsequent hydrocephalus successfully treated with a temporary lumbar drain.

### Case Description

**Figure 1:**
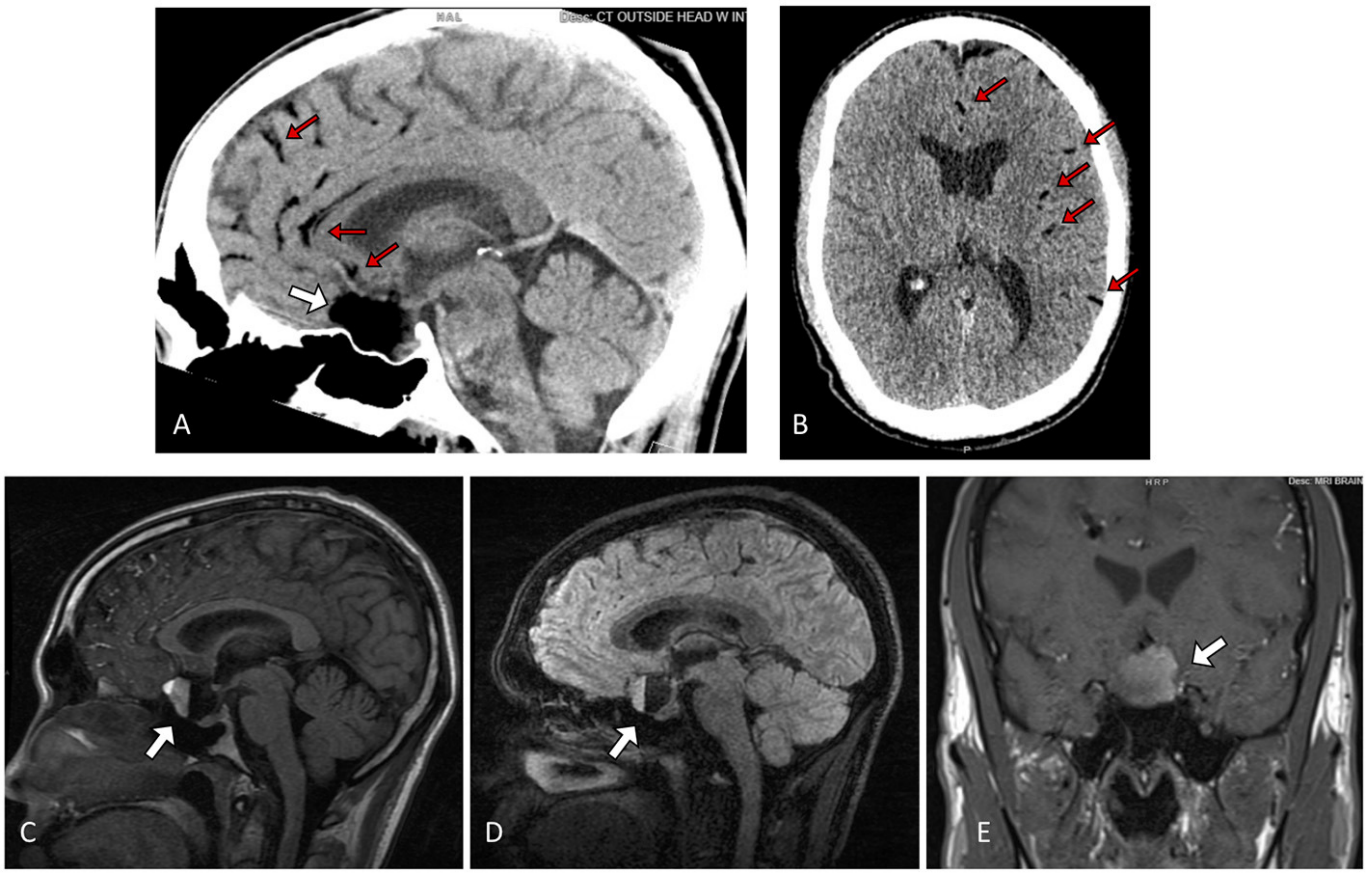
Sagittal **(A)** and Axial **(B)** non contrast Head CT showing a hypodense lesion in the sella/suprasellar region (white arrow), with associated scattered hypodense globules along the parasagittal sulci and left sylvian fissure (red arrows) **(C)** Sagittal T1 fluid-attenuated inversion recovery (FLAIR), **(D)** 3D T2 fast spin-echo (FSE), and **(E)** Coronal T1-weighted MRI showing a ruptured sellar dermoid cyst with pathognomonic blood-fat-CSF level (white arrow).

A 30-year-old man with a history of migraines presented to the emergency department with 2 days of frontal headaches, photophobia, nausea, vomiting, and acute vision loss in the left eye. The neurological exam was within normal limits, and no focal neurologic deficits were elicited. Non-contrast CT of the head revealed a hypodense, fat-containing suprasellar mass. Brain MRI with contrast showed a non-enhancing 2.5 × 2.3 × 2.3 cm suprasellar mass with fluid-fluid level and associated mass effect on the optic chiasm, pituitary gland, and inferior medial aspect of the bilateral frontal lobes. Multiple areas of scattered foci of increased T1 signal within the left and right convexity sulci and sylvian fissure were noted, suggestive of disseminated subarachnoid fat due to a ruptured dermoid cyst (Figure 1).

On physical examination, the patient had an afferent dilated left pupil, left ptosis, and diplopia. The patient was noted to have a low grade fever to a maximum temperature of 38.2°C, and white blood cell count was elevated to 18.10 K/uL with neutrophilic predominance. Sodium was 135 mmol/L, and lactic acid was 4.6 mmol/L. A lumbar puncture revealed cloudy cerebrospinal fluid (CSF) with 60 mg/dL glucose, 144.3 mg/dL protein, and 2,640 nucleated cells/uL with 97% neutrophils. CSF culture and gram stain were negative. Opening pressure was >55 cmH_2_O, and closing pressure was 44 cmH_2_O following removal of 8cc of CSF. Consequently, a right frontal EVD was emergently placed with improvement of the mental status. Concomitantly, the patient received a bolus of 10 mg Dexamethasone IV.

Because of persistent poor vision in the left eye, the patient was taken to the operating room for a left pterional craniotomy for evacuation and decompression of the sellar lesion. Upon dissection of the Sylvian fissure, the arachnoid space was notably adherent with disseminated fatty appearing material throughout. The arachnoid surrounding the optic nerves was similarly adherent to the nerve, and the yellowish tan cyst was seen compressing the nerves and optic chiasm. The cyst was drained of tan-white, friable, amorphous material and with adequate decompression of optic apparatus. The capsule of the cyst was peeled off of the most anterior and inferior structures, but part of the capsule was completely adherent to both optic nerves and the infundibular stalk posteriorly, and this part of the capsule was left in place to avoid further damage to the neurovascular structures.

Postoperatively, the vision in the left eye dramatically improved; however, over the following 3 days, attempts at removing the EVD failed twice due to increased ICP and symptom re-occurrence.

A lumbar drain was placed on postoperative day 3 and leveled at 5–10 cc/hr as an attempt to drain particulate from the subarachnoid spaces. The CSF from the subarachnoid space was cloudy and contained 5.3 mmol/L lactate, 54 mg/dK glucose, 240.5 mg/dL protein, and 415 nucleated cells/uL with 98% neutrophils. Culture and gram stain were negative. Upon insertion of the lumbar drain, the EVD was raised to 20 cmH_2_O so that most of the drainage occurred *via* the lumbar drain.

On postoperative day 6, CSF from the lumbar drain had significantly improved and contained 7.2 mg/dL protein, 90 mg/dL glucose, and 11 nucleated cells/uL and the EVD was clamped. Forty-eight hours later, the lumbar drain was also clamped for the next 24 h, at which point both drains were removed upon evidence of stably normal ICP and no clinical or radiological evidence of hydrocephalus.

The patient was discharged home the following day.

**Figure 2:**
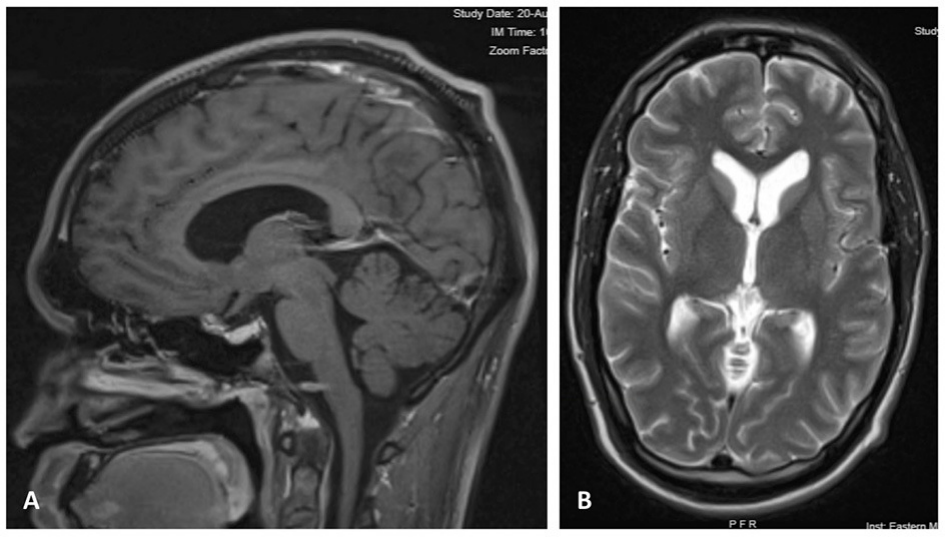
**(A)** Contrast-enhanced Sagittal T1 image 6 months after craniotomy, showing decompression of the sellar region with resolved mass effect over the optic pathway. **(B)** Axial T2 MRI at 6 months after surgery, showing normal size of ventricles and sulci.

At a 2-month follow-up, the patient was doing well, headaches and diplopia resolved, and minimal residual blurry vision in the left eye continued to improve. A repeat head CT showed no evidence of recurring hydrocephalus. By the 6-month follow-up, all neurological and visual deficits had resolved, and a repeat MRI of the brain showed stable sella decompression and stable ventricular size (Figure 2).

### Pathology

**Figure 3:**
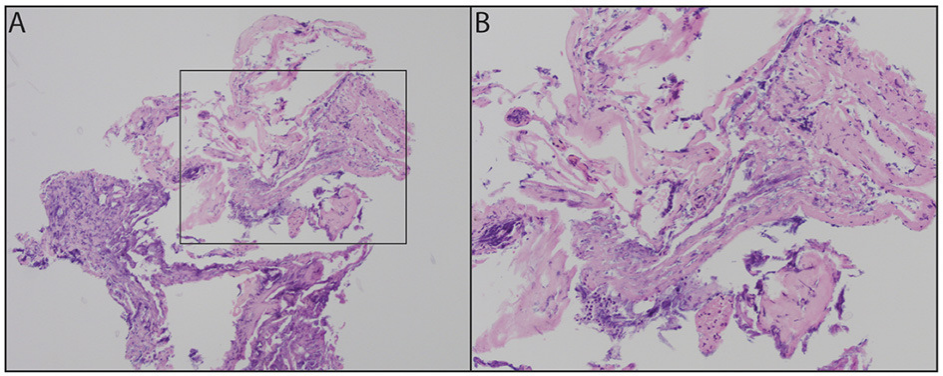
Fibrous tissue with acute inflammatory infiltrate and cauterization artifacts at 100x **(A)** and 200x **(B)**.

Analysis of the capsule demonstrated inflamed fibrous tissue compatible with cyst lining (Figure 3), and the contents contained material consistent with fat and blood.

## Discussion

Intracranial dermoid cysts account for 0.3% of all brain tumors (4). They are congenital, non-neoplastic tumors that arise from inclusion of ectodermally committed cells at the time of neural tube closing around the 3rd to 5th weeks of embryogenesis. This explains why they typically appear close to the midline (3). They have an outer capsule of dense fibrous connective tissue lined with stratified squamous epithelium and can contain varying amounts of ectoderm derivatives, including apocrine, sweat, and sebaceous glands as well as hair follicles, squamous epithelium, and possibly teeth (3, 11). The suprasellar, parasellar, frontobasal region, or posterior fossa are the most frequent locations (11), typically presenting in the second or third decades of life (3).

Through accumulation of desquamation products and sebaceous secretions into the cystic cavity, dermoid cysts slowly grow. They may be identified incidentally on CT or MRI scans, or may present symptomatically with headaches, seizures, or rarely olfactory delusions (3).

Although it was initially assumed that rupture of intracranial dermoid cysts was always fatal, later cases disproved this (12). Surgical removal with extensive rinsing of subarachnoid space during operation is generally required (12). If the capsule is strongly adherent to neurovascular structures, subtotal resection should be considered to avoid vascular complications (8). Recurrence after subtotal removal is extremely rare, but incompletely resected cysts should be followed routinely (1, 9, 12). Surgical options for decompression, especially in cases of sellar/suprasellar lesions, includes endoscopic endonasal approaches (EEA) vs. traditional craniotomy (13–16). In this case, an open craniotomy was preferred due to the concomitant hydrocephalus and elevated ICP, and the resultant higher risk of postoperative CSF leak associated to EEA.

During surgery, it is not always possible to remove disseminated fat droplets, which can be diffuse and widespread. Over time, these do not appear to be resorbed and persist for years after time of rupture (8). The literature presents conflicting evidence on whether or not this is a problem. Liu et al. followed five patients with surgically removed ruptured dermoid cysts with serial MRI scans and clinical examinations and noted that extensive disseminated fat particles did not demonstrate progression or movement or new neurological deterioration. They determined that it is not necessary to remove the fat droplets during surgery (8). However, some studies conclude that presence of fat in the ventricles and subarachnoid spaces produces many complications such as aseptic meningitis, hydrocephalus, vasospasm, and cerebral ischemia (10). Some authors argue that communicating hydrocephalus may be a consequence of aseptic meningitis/arachnoiditis from disseminated fat particles (12, 16). Martin et al. reported a case of obstructive hydrocephalus as a result of ruptured dermoid cyst, which was credited to intraventricular fat (17). A handful of cases in the literature have documented hydrocephalus following intracranial dermoid cyst rupture (Table 1). Some of these studies at least partially credited disseminated fat for playing a role in the development of hydrocephalus (8, 9, 11, 19, 20, 22). In a report of a ruptured right parasellar dermoid cyst that led to convulsive seizures a few years after removal, Fukui commented that the symptoms were likely due to residual fat particles in the Sylvian cistern, and concluded that these should be removed as much as possible at the time of surgery (23). Orakcioglu et al. discussed two cases with postoperative fatty particles. In one patient, the particles obstructed intraventricular pathways and led to obstructive hydrocephalus, but in another, hydrocephalus never developed (18). Karabulut and Oguzkurt reported a case of tetraventricular hydrocephalus after intracranial dermoid cyst rupture credited partially to subarachnoid and cisternal fatty material (11).

**Table 1:** Details of literature review of patients who presented with hydrocephalus post intracranial dermoid cyst rupture.

**References**	**Demographics**	**Location of cyst**	**Treatment**
Karabulut and Oguzkurt (11)	26-year-old male	Right interpeduncular cistern	Not discussed
Liu et al. (8)	35-year-old male	Left perisylvian with suprasellar extension	VP shunt
Liu et al. (8)	36-year-old male	Pineal region with parieto-occipital expansion	VP shunt
Orakcioglu et al. (18)	33-year-old male	Intrasellar, roof of orbit, right petrous bone, intraventricular	VP shunt
Esquenazi et al. (9)	47-year-old female	Floor of anterior cranial fossa extending superiorly along the falx	EVD and VP shunt
Murrone et al. (19)	43-year-old female	Crista galli with left frontal region expansion	EVD and VP shunt
Wani et al. (20)	30-year-old male	Cerebellar vermis	VP shunt
Bishnoi et al. (21)	48-year-old male	Right frontal horn	EVD
Shashidhar et al. (22)	32-year-old male	Right Meckel's cave	VP shunt
Shashidhar et al. (22)	42-year-old male	Suprasellar	VP shunt

*EVD, external ventricular drain; VP, ventriculoperitoneal*.

When postoperative hydrocephalus does develop in these cases, the most common treatment is placement of a ventriculoperitoneal (VP) shunt (8, 9, 18) (Table 1). This leads to symptomatic improvement and prevention of brain damage (23) but makes the patient shunt dependent, usually for life. In the case presented, as the patient continued to have non-obstructive hydrocephalus despite proper ventricular CSF drainage *via* an EVD, placement of a lumbar drain cleared the disseminated fatty particles from the patient's subarachnoid space, facilitating the resolution of the hydrocephalus. The ventricular size returned to normal, and there was no recurrence of hydrocephalus.

This is the first case reported in literature in which hydrocephalus after surgically removed ruptured intracranial dermoid cyst was resolved with a lumbar drain. We hypothesize that, differently from an EVD, a lumbar drain directly addresses the reason for the obstructive hydrocephalus—which is engorgement of the subarachnoid space with fat droplets and inflammatory cells—by directly washing out the affected anatomical space. This technique has also been supported in cases of postoperative intraventricular hemorrhage with persisting communicating hydrocephalus (24–28). Placement of a temporary lumbar drain for a 5–7 days course, as it is in the authors' experience, is a safe and relatively low-invasive procedure, usually preformed at the bedside.

It cannot be excluded that the combination of both the EVD and the lumbar drain have determined the resolution of the hydrocephalus. However, it remains evident that until the placement of the lumbar drain, it had been impossible to clamp the EVD without incurring in increased ICP and clinical manifestations of raised ICP.

## Conclusion

Intracranial dermoid cysts are uncommon, and rupture is a rare event. Surgical intervention is the mainstay of treatment, and subarachnoid spaces should be copiously irrigated at the time of surgery in order to clear out any residual dermoid contents. Despite this, disseminated fat particles often persist throughout the subarachnoid spaces. In the case of a ruptured suprasellar dermoid cyst, postoperative communicating hydrocephalus developed due to persistent fat particles in the subarachnoid spaces. In this case we found that temporary CSF drainage *via* a lumbar drain likely contributed to the resolution of the hydrocephalus by clearing the subarachnoid space of persistent fatty particles. Although the success of one case is not sufficient to suggest a new protocol, this relatively low-impact procedure could be considered in similar cases with refractory ICP before submitting the patient to permanent CSF diversion.

## Data Availability Statement

The original contributions presented in the study are included in the article/supplementary material, further inquiries can be directed to the corresponding author/s.

## Ethics Statement

Ethical review and approval was not required for the study on human participants in accordance with the local legislation and institutional requirements. The patients/participants provided their written informed consent to participate in this study. Written informed consent was obtained from the individual(s) for the publication of any potentially identifiable images or data included in this article.

## Author Contributions

All authors participated in the clinical care of this patient and/or drafting of the manuscript.

## Conflict of Interest

JB has an equity position in Avidea Technologies, Inc., which is commercializing polymer-based drug delivery technologies for immunotherapeutic applications and has an equity position in Treovir LLC, an oHSV clinical stage company and is a member of the POCKiT Diagnostics Board of Scientific Advisors. The remaining authors declare that the research was conducted in the absence of any commercial or financial relationships that could be construed as a potential conflict of interest.

## Publisher's Note

All claims expressed in this article are solely those of the authors and do not necessarily represent those of their affiliated organizations, or those of the publisher, the editors and the reviewers. Any product that may be evaluated in this article, or claim that may be made by its manufacturer, is not guaranteed or endorsed by the publisher.
